# Tuning Solvation Dynamics of Electrolytes at Their Eutectic Point Through Halide Identity

**DOI:** 10.3390/molecules30102113

**Published:** 2025-05-09

**Authors:** Rathiesh Pandian, Benworth B. Hansen, Giselle de Araujo Lima e Souza, Joshua R. Sangoro, Steven Greenbaum, Clemens Burda

**Affiliations:** 1 Department of Chemistry, College of Arts and Sciences, Case Western Reserve University, Cleveland, OH 44106, USA; rrp45@case.edu; 2 William G. Lowrie Department of Chemical and Biomolecular Engineering, The Ohio State University, Columbus, OH 43210, USA; hansen.663@buckeyemail.osu.edu (B.B.H.); sangoro.1@osu.edu (J.R.S.); 3 Department of Physics & Astronomy, Hunter College of the City University of New York, New York, NY 10065, USA; giselle.souza@hunter.cuny.edu (G.d.A.L.e.S.); sgreenba@hunter.cuny.edu (S.G.)

**Keywords:** deep eutectic solvents, femtosecond transient absorption spectroscopy, solvation dynamics, solvent structure, Reichardt’s dye betaine-30, *E*_T_(30) polarity, differential scanning calorimetry, choline halides, self-diffusion, pulsed-field gradient NMR spectroscopy

## Abstract

Deep eutectic solvents (DESs) are regarded as highly promising solvent systems for redox flow batteries. DESs, composed of choline halides (ChX, X = F^−^, Cl^−^, Br^−^, I^−^) and ethylene glycol (EG), exhibit distinct physicochemical properties at their eutectic points, including halide-dependent phase behavior, viscosity, polarity, conductivity, and solvation dynamics. In this study, we investigate the effects of the halide identity on the solvation properties of ChX:EG mixtures at varying mol % of ChX salt content. The solvatochromic polarity based on *E*_T_(30) measurements indicates higher polarity for larger halides (I^−^ > Br^−^) than for smaller halides (Cl^−^ > F^−^), which exhibit larger compensating solvation shells. The ionic conductivity follows the trend of the solvent fluidity (the inverse of the viscosity), namely ChCl > ChBr > ChI > ChF, influenced by the ion mobility and solvodynamic radii. Measurements of the liquidus temperatures (*T*_L_) reveal that the system with ChCl exhibits the deepest eutectic point (at ~20 mol % ChCl), while ChBr and ChI have shallower minima at ~10 mol % ChBr and ~3 mol % ChI, respectively. ChF does not display a eutectic transition but instead appears to readily supercool at salt concentrations above 30 mol % ChF. Consistent with the phase transition measurements, femtosecond transient absorption spectroscopy shows that in the ChCl system, the solvation dynamics become faster with an increasing salt concentration up to ~16.67 mol %, after which the dynamics slow down with further increases in the salt content. The ChF-based system exhibits similar behavior, though with slower dynamics. In contrast, the solvation dynamics of the systems containing ChBr and ChI monotonously slow down with an increasing salt concentration, in agreement with the phase transition measurements, which show that the eutectic points occur at low salt concentrations. These measurements suggest that the solvent composition and, in particular, the identity of the halide anion play a significant role in the solvation behavior of these ethylene-glycol-based DESs, offering a foundation for tuning the DES properties for specific applications.

## 1. Introduction

With the rise in industrialization and population growth, the global demand for energy continues to increase. Currently, traditional fossil fuels dominate the energy sector but continue to become increasingly costly [1]. There has been a strong push toward the development and integration of renewable energy technologies, such as solar, wind, and hydroelectric power. While these renewable energy procurement methods can be sustainable, they unfortunately suffer from major issues in terms of their intermittency and unpredictability; factors such as the weather conditions, climate, and time of day seriously reduce their reliability. Due to this, it is necessary to develop energy storage technologies capable of storing large amounts of excess energy and releasing it when needed [2,3,4].

A type of battery system that could potentially handle the high demands of grid-scale energy storage are redox flow batteries (RFBs). They operate by circulating liquid electrolytes containing dissolved redox-active species through a flow cell where oxidation and reduction reactions occur. The use of liquid electrolytes enables a long cycle life, as the charge transfer occurs in a liquid medium rather than a solid electrode, reducing the degradation. Compared to lithium-ion battery systems, RFBs offer enhanced safety and stability and have a much lower risk of a thermal runaway event [2,3,5]. These advantages make RFBs possible candidates for grid-scale applications where long-term reliability and stability are necessary. Despite their potential advantages, current RFB systems, which are primarily aqueous, face several limitations. Due to their narrow electrochemical window, aqueous RFBs can still be improved in terms of the operational voltage and energy densities [6]. To overcome these challenges, researchers are investigating alternative non-aqueous electrolyte systems that can improve the electrochemical stability window to ultimately enhance the battery performance and increase the energy storage capacity.

A promising class of alternative electrolytes for RFBs are deep eutectic solvents (DESs). DESs are solvent mixtures that are most commonly formed by combining a hydrogen-bond acceptor (HBA) and a hydrogen-bond donor (HBD). Strong hydrogen-bonding interactions between these components can lead to eutectic melting point depressions, allowing the mixture to exist as a liquid at room temperature [7,8,9,10,11]. They were first introduced in 2003 by Abbott et al., who found that a mixture of the powdered solids choline chloride (ChCl; melting point ≈ 303 °C) and urea (melting point = 134 °C) at a 1:2 molar ratio resulted in a mixture with a melting point of 12 °C, making it a liquid at room temperature, often referred to as “reline” [10]. Since then, DESs have attracted interest due to their potentially low cost, ease of preparation, biodegradability, low toxicity, and tunability [10,12,13]. One of the key advantages of DESs is the potential for broader electrochemical windows, which enables higher voltage operation compared to aqueous electrolytes. Additionally, DESs exhibit high solubility for redox-active species, which can improve the electrolyte performance by allowing greater charge storage capacity. In the context of RFBs, the use of DES-based electrolytes could overcome the limitations of conventional aqueous systems while providing high safety and scalability at a relatively low cost [14]. Despite their potential, however, DESs tend to exhibit relatively high viscosities and mediocre conductivities, which impact the charge transfer kinetics and, consequently, the overall battery efficiencies. Instead of just optimizing specific mixtures, this study focuses on identifying general design principles to help select components that lower the viscosity while keeping the good electrochemical properties. Choline halides in ethylene glycol (EG) serve as a model system to understand how different halides influence key behaviors, which can guide the development of better DESs for large-scale RFB applications.

Over the past two decades, numerous DES systems have been investigated. One such example is the 1:2 molar ratio (33.33 mol % ChCl) mixture of ChCl and EG, referred to as “ethaline” [7]. Over the years, ChCl has been commonly used as an HBA in the formation of DESs [11]. However, there are significantly less studies available involving choline fluoride (ChF), choline bromide (ChBr), or choline iodide (ChI). The halide used should contribute significantly to determining properties such as the viscosity, polarity, ionic conductivity, and solvation dynamics. Smaller halide anions, such as fluoride (F^−^), exhibit strong hydrogen-bonding interactions. In contrast, larger halides, such as bromide (Br^−^) and iodide (I^−^), exhibit weaker hydrogen bonding [15]. This is in good agreement with the findings of Peris et al., who determined using ^1^H NMR that the H⋯F^−^, H⋯Cl^−^, H⋯Br^−^, and H⋯I^−^ bond strengths are 21.76, 8.79, 7.53, and 5.44 kJ mol^−1^, respectively [16,17]. Therefore, a systematic study of each halide ion (F^−^, Cl^−^, Br^−^, and I^−^) in choline-based DESs can help classify how variations in the hydrogen-bonding strength, charge density, and ionic radius influence the solvent behavior across the halide series. Understanding these effects is necessary for designing optimized DES-based electrolytes for energy storage applications.

While ChCl-based DESs have been studied extensively, there is very little information on ChF-, ChBr-, and ChI-based DESs. Shen et al. [15] found, through FTIR spectroscopy and DFT calculations, that in mixtures of tetrabutylammonium halides (TBAX, X = Cl, Br, I) and EG (at a 1:8 TBAX:EG molar ratio), the O–H stretch frequency was 3318, 3322, and 3334 cm^−1^ for the TBACl:EG, TBABr:EG, and TBAI:EG mixtures, respectively. In addition, the O–H bond lengths in EG were computed to be 0.990, 0.987, and 0.984 Å for the TBACl:EG, TBABr:EG, and TBAI:EG mixtures, respectively. The corresponding Cl^−^⋯HO, Br^−^⋯HO, and I^−^⋯HO hydrogen bond lengths were computed to be 2.17, 2.36, and 2.65 Å, respectively. These results indicate that as the halide increases in size, the O–H bond in EG gets shorter and the X^−^⋯HO hydrogen bond lengthens [15]. Recently, in ChX:EG (1:9) mixtures, Prado et al. found that the ChF system exhibits the highest electrochemical stability window of 2.14 V, followed by 1.84, 1.62, and 1.18 for the ChCl:EG, ChBr:EG, and ChI:EG systems, respectively [18]. Boogaart et al. showed that in iodide-based DES electrolytes for dye-sensitized solar cells, the halide identity significantly impacts the viscosity and device performance, with lower hydrogen-bonding affinity halides like iodide enabling higher photocurrents and efficiencies. Notably, DESs incorporating the redox couple directly into the electrolyte matrix (such as those based on ChI) eliminate the diffusion limitations and enhance the photovoltaic efficiency, conveying the importance of tuning the halide composition for optimized fluidity and electrochemical function [19].

Recent molecular dynamics simulations by Lane et al. of DESs composed of L-leucic acid and quaternary ammonium halide salts revealed that the hydrogen bonding between the HBD and the halide, the cation and the halide, and the HBD and the cation are key interactions across a variety of cation structures. Larger cations form more persistent hydrogen bonds, particularly between the ammonium hydrogens and the HBD carbonyl group, facilitating DES formation. Systems with smaller anions (such as Cl^−^ in the work by Lane et al.) exhibit stronger and more persistent hydrogen bonding compared to those with larger anions (I^−^). The domain analysis also indicated nanoscale heterogeneity, with more homogeneous mixtures formed by smaller cations. Their findings emphasize that both the cation size and halide identity significantly influence the hydrogen-bonding networks and the likelihood of stable DES formation [20].

We recently synthesized a ChF:EG mixture at a 1:2 ratio and compared it to ethaline (1 mol ChCl:2 mol EG). We found that in the 1:2 ChF:EG mixture, ^19^F NMR revealed there to be two distinct fluoride peaks, concluding that some of the fluoride anions (~15%) are coordinated to choline, while the remaining are freely solvated in EG [21,22]. This feature (for chloride) is not found in ChCl systems [23]. Furthermore, we found through self-diffusion measurements attained by pulsed field gradient NMR that the EG is the faster diffusing component compared to choline [22]. According to D’Agostino et al., in ChCl:EG mixtures, the same trend of HBD EG diffusion being fastest is also observed [24].

Femtosecond transient absorption (fs-TA) spectroscopy has been used to probe the solvent properties in this subset of choline halide + EG-based DESs by measuring the solvent relaxation dynamics. The aim of this process is to provide insights into the molecular environment surrounding the solvated species. To this end, we selected Reichardt’s dye, also known as betaine-30 (B30) [25], for its properties as a solvatochromic probe, which makes it responsive to changes in the solvent polarity and relaxation dynamics. Once photoexcited, B30 undergoes an intramolecular charge transfer (CT) process where the electron density shifts from the phenolate group to the pyridinium ring. This results in a significant change in the dipole moment of 21 Debye from B30’s more polar ground state (+15 Debye) to its more nonpolar excited state (−6 Debye) [26], making B30 highly sensitive to the surrounding solvent environment [27,28,29,30,31,32,33,34,35,36,37]. In addition, the solvent reorganization response following B30’s photoexcitation is primarily governed by the relaxation dynamics of the surrounding solvent itself rather than B30. As a result, the rate-limiting step is the reorganization dynamics of the solvent, providing an opportunity to directly measure the solvation dynamics [38,39]. In more polar solvents, the UV-Vis absorption band of B30 exhibits a blueshift due to the stronger stabilization of its ground state over its excited state. This solvatochromic shift allows for a quantitative assessment of the solvent polarity using a scale referred to as the *E*_T_(30) polarity scale, also developed by Reichardt [25,33].

In this work, we investigate DES mixtures of choline halides (ChF, ChCl, ChBr, ChI) with EG across a range of liquid compositions, focusing on their liquidus temperature, viscosity, density, polarity, conductivity, and solvation dynamics. We assess how the halide identity influences these key properties by using solvation dynamics measurements to provide key insights into the solvent relaxation and charge transport characteristics. Additionally, we examine the liquidus temperatures to understand the phase and thermal stability, as well as the density trends to explore the structural and packing effects within the mixtures. By establishing correlations between the halide selection and the solvent properties, this study aims to improve the understanding of how DESs can be tailored for energy storage applications and provide a foundation for optimizing their performance in large-scale RFB battery systems. While direct electrochemical testing in an RFB is beyond the scope of this study, the molecular-level insights into the solvation dynamics and transport properties presented here are intended to inform the rational design of DES-based electrolytes for future RFB applications.

## 2. Results

### 2.1. Solvent Relaxation Dynamics

Fs-TA pump–probe measurements were performed to elucidate the solvent relaxation dynamics following a laser-pulse-induced charge transfer (CT) of the reporter molecule Reichardt’s dye B30. The transient absorption signal, TA(*t*, *λ*), was fitted using a biexponential function, as shown in Equation (1).

TA(*t*, *λ*) = *A*_1_ exp(−*t*/*τ*_1_) + *A*_2_ exp(−*t*/*τ*_2_) (1)

In Equation (1), the fitting parameters include the amplitudes (*A*_1_ and *A*_2_) and time constants (*τ*_1_ and *τ*_2_), with R^2^ values higher than 0.99. In DES solvent mixtures, *τ*_1_ is the faster component and is generally attributed to the ethylene glycol (EG) reorientation dynamics on the picosecond timescale. The time constant *τ*_2_ captures the slower component and generally represents the response of choline, which we have previously assigned to be the slowest species in self-diffusion measurements [22,24,40,41].

**Figure 1:**
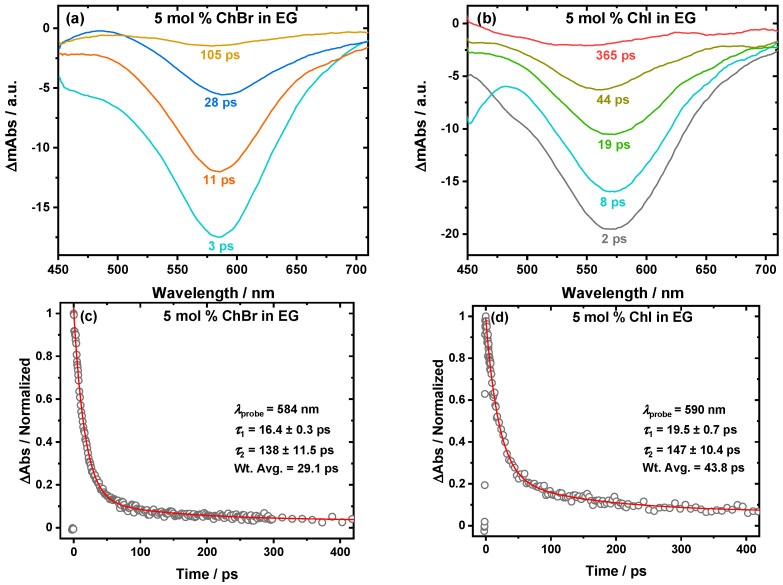
Sample fs-TA spectra of B30 dissolved in (**a**) 5 mol % ChBr in EG and (**b**) 5 mol % ChI in EG. The negative signal corresponds to ground-state bleaching. The bleach becomes less negative as the delay time, which is in picoseconds (ps), increases. The corresponding biexponential kinetic decay traces are shown in panels (**c**,**d**), respectively. Fitting was performed over a spectral range of *λ*_fitted_ = 500–600 nm. *T* = 298 K.

Sample transient spectra are shown in Figure 1a,b at 5 mol % ChBr in EG and 5 mol % ChI in EG, respectively. The negative signal arises from ground-state bleaching, which is most pronounced immediately following photoexcitation. As the system relaxes back to the ground state, the transient bleach signal diminishes in magnitude (i.e., becomes less negative) with an increasing delay time [42]. From these transient spectra, kinetic decay traces can be derived. This is shown in Figure 1c,d for 5 mol % ChBr in EG and 5 mol % ChI in EG, respectively. The relaxation time constants are derived by the biexponential fitting of the kinetic decay traces using Equation (1).

**Figure 2:**
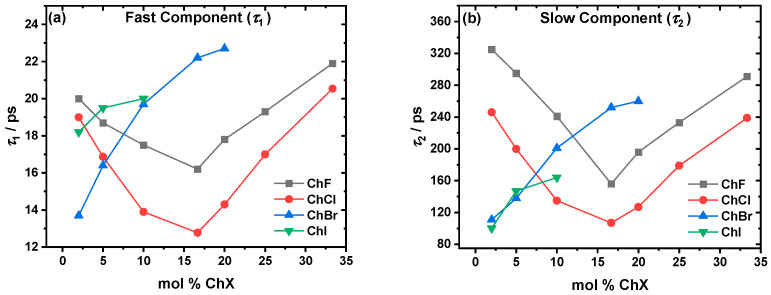
(**a**) Fast, τ₁, and (**b**) slow, τ_2_, relaxation components from the biexponential fitting of the fs-TA data for the ChF (gray), ChCl (red), ChBr (blue), ChI (green), and EG mixtures. There are clear minima in both components for ChF and ChCl, while the ChBr and ChI systems show a generally continuous slowing down as a function of the increasing mol % ChX. *λ*_fitted_ = 500–600 nm. Lifetimes for pure EG are omitted from the plots but are present in the SI. See Tables S5–S8 for the numerical results and uncertainties. All the errors were kept within ±10%. *T* = 298 K.

The parameters (fast, *τ*_1_, Figure 2a; slow, *τ*_2_, Figure 2b_)_ determined experimentally through fs-TA are dependent on the halide and mol % of ChX. The dynamics for ChF and ChCl speed up, reaching a minimum at ~16.67 mol % ChF/ChCl, and then proceed to slow down. Overall, ChF is slower than ChCl. On the other hand, the dynamics of the systems with larger halides, ChBr and ChI, trend in a completely different way. For both these systems, the dynamics tend to slow down continuously with the addition of ChBr and ChI.

### 2.2. Liquidus Temperatures

**Figure 3:**
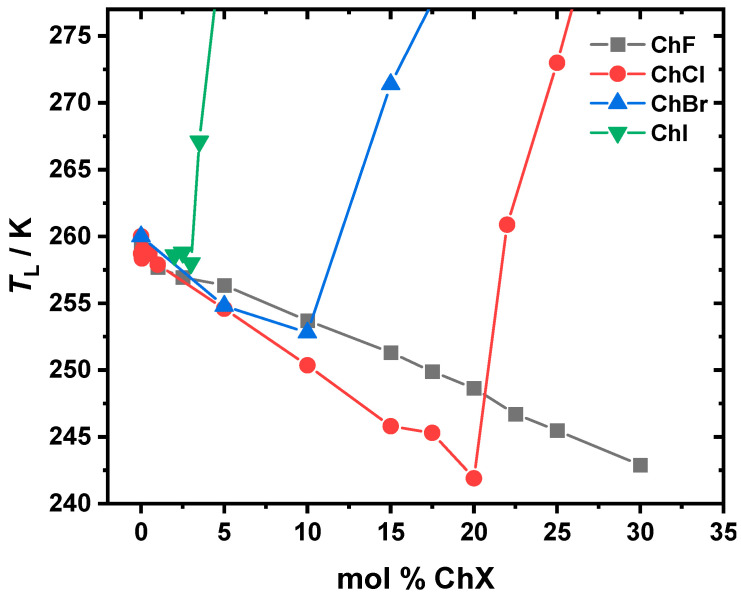
Liquidus temperatures (*T*_L_) as a function of the mol % ChF (black squares), ChCl (red circles), ChBr (blue triangles), and ChI (green) in EG. The *T*_L_ decreases continuously for ethalineF up to 30 mol % ChF, where the mixture transitions into a glassy state. For ethalineCL, the *T*_L_ decreases until ~20 mol % ChCl, corresponding to the eutectic composition, and then increases with further ChCl addition. In the ChBr and ChI systems, the minima are observed at ~10 mol % and ~4 mol %, respectively, indicating their eutectic compositions. The uncertainty in terms of the temperature is ±0.5 K.

DSC was conducted to determine the liquidus temperatures (*T*_L_). All the systems experience a depression of the *T*_L_ as a function of the increasing mol % ChX, as depicted in Figure 3. For pure EG, the *T*_L_ = (*T_m_*) = 260.2 K. As ChI is added, there is a suppression of *T*_L_ until ~4 mol % ChI, at which point the *T*_L_ is 267 K. The ChBr has a deeper *T*_L_ suppression, with the minimum occurring at 253 K at ~10 mol % ChBr. The *T*_L_ suppression for the ChCl system is even deeper, reaching to 242 K at ~20 mol % ChCl. Finally, although the ChF system appears to exhibit an even deeper suppression of *T*_L_, this behavior is not directly observed, as the system transitions into a supercooled “glassy” liquid state beyond 30 mol % ChF, without an explicitly detectable melting point. These minima show the same composition trends as the dynamic enhancements observed in Figure 2 and demonstrate that smaller halides lead to deeper eutectic points. Please see the Section 3. below for a full description of our interpretation of Figure 3.

### 2.3. Viscosity, Ionic Conductivity, and Density

**Figure 4:**
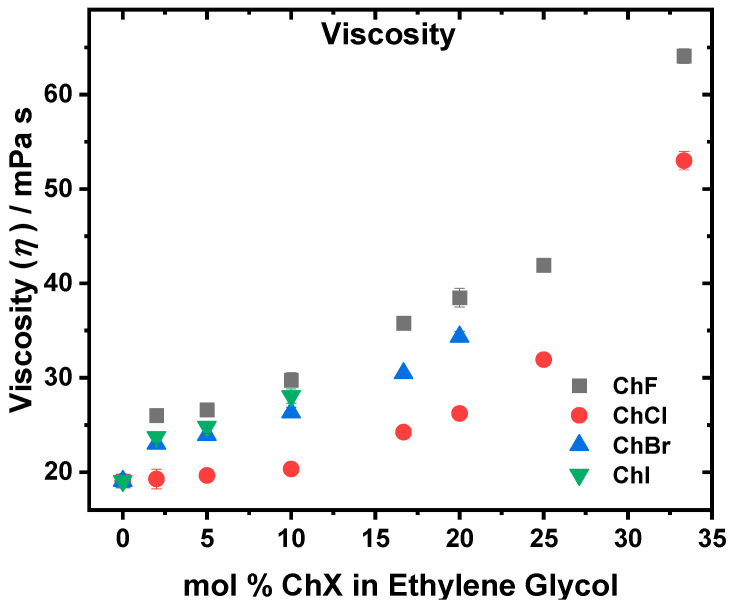
Viscosity as a function of the mol % ChF (gray), ChCl (red), ChBr (blue), and ChI (green) in EG. The viscosities increased as a function of the increasing mol % ChX. The ChF system was the most viscous, followed by ChI and then ChBr (which both had similar viscosities), and finally ChCl, which was the least viscous. See Tables S5–S8 for the numerical results and uncertainties. *T* = 298 K.

The viscosity (*η*), ionic conductivity (*σ*), and density (*ρ*) of the ChX:EG systems at varying mol % ChX were measured. The viscosity as a function of the mol % ChX in EG is shown in Figure 4. All the systems show an increase in viscosity as ChX is added to the system. The most viscous system is ChF, followed closely by ChI, ChBr, and then ChCl. Due to solubility limitations, we stopped the ChBr and ChI preparations at 20 and 10 mol %, respectively.

**Figure 5:**
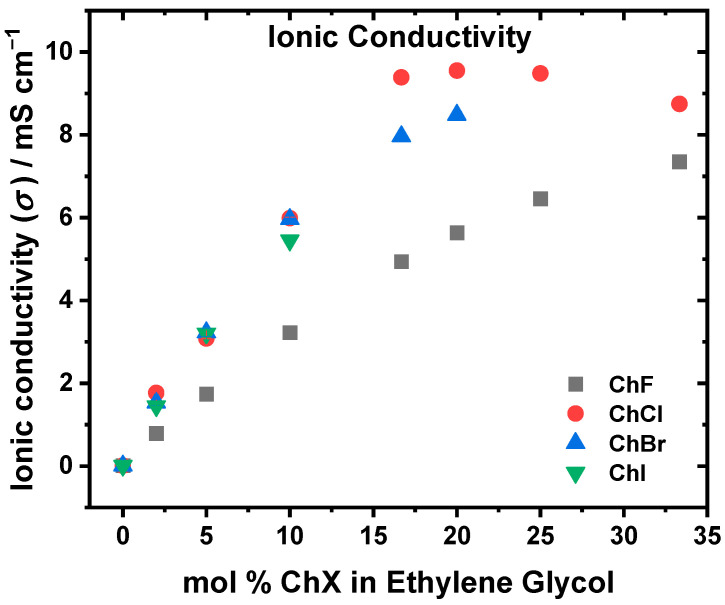
Ionic conductivity as a function of the mol % ChF (gray), ChCl (red), ChBr (blue), and ChI (green) in EG. The conductivities increased as a function of the increasing mol % ChX. The most conductive system was ChCl, where the peak conductivity at ~20 mol % ChCl correlates with the eutectic composition. The least conductive system was ChF, due to the stronger F⁻⋯OH interactions between ChF and EG, which result in a more rigid hydrogen bonding network, and the generally ordering effect of the dissolved fluoride ion. See Tables S5–S8 for the numerical results and uncertainties. *T* = 298 K.

The ionic conductivities for all the systems are shown in Figure 5 and follow the following trend: ChCl > ChBr > ChI > ChF. Interestingly, for ChCl, ChBr, and ChI, the conductivities are very similar until as much as 5 mol % salt addition. Beyond this, the conductivity of ChI lags slightly at 10 mol % while ChCl and ChBr remain close. At 16.67 mol %, ChCl starts to have the highest ionic conductivity, maxing out at ~20 mol % ChCl. The viscosities and conductivities for the ChCl system are in good agreement with the literature values [41,43,44].

**Figure 6:**
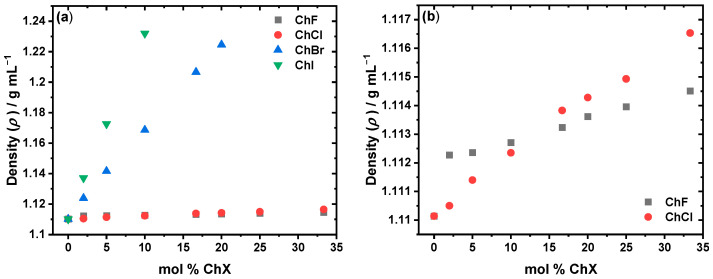
Density as a function of the mol % ChF (gray), ChCl (red), ChBr (blue), and ChI (green) in EG for (**a**) all the halide mixtures and (**b**) specifically the ChF and ChCl mixtures. ChBr and ChI exhibit the highest densities, with a pronounced increase as the mol % ChX increases, due to their larger halide mass. ChCl shows a more gradual density increase, while ChF starts out slightly denser than ChCl at low mol % (2–10%) but becomes less dense beyond ~16.67 mol %. All the measurements were taken at room temperature (*T* = 298 K). The density for the ChCl-based solvent system has been previously reported [41]. See Tables S9–S12 for the numerical values across 298.15–323.15 K.

The densities as a function of the mol % ChX at room temperature (298 K) are presented in Figure 6. Figure 6a compares all the halides, showing that the ChI and ChBr systems have significantly higher densities than ChCl and ChF. Between 3 and 10 mol % ChX, the density trend is as follows: ChI > ChBr > ChF > ChCl. However, at 16.67 mol % and higher salt concentrations, the order shifts to ChI > ChBr > ChCl > ChF. Initially, the ChF system is denser than ChCl, but at 16.67 mol % and higher, ChCl surpasses ChF in density (Figure 6b).

### 2.4. Polarity

The polarities of the systems were experimentally determined using the *E*_T_(30) polarity scale, as established by Reichardt, using Equation (2) [25,27,30,35,45].

*E*_T_(30) (kcal mol^−1^) = *h c N*_A_ *ν*_max_ = 28591/*λ*_max_ (2)

**Figure 7:**
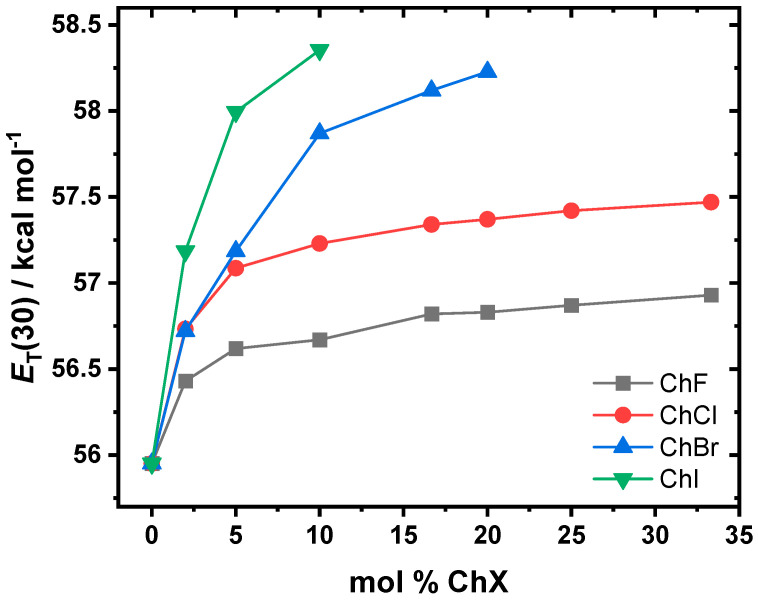
*E*_T_(30) polarity as a function of the mol % ChF (gray), ChCl (red), ChBr (blue), and ChI (green) in EG. The polarity is highest for the ChI-based system, followed by ChBr, ChCl, and ChF. See Tables S1–S4 for the numerical values.

In Equation (2), *h* is Planck’s constant, *c* is the speed of light, *N*_A_ is Avogadro’s number, and *ν*_max_, *λ*_max_ correspond to the wavenumber and wavelength, respectively, at the absorption peak of B30 for its longest-wavelength CT transition upon its photoexcitation (see Figure S1 for a depiction of this process). As the solvent polarity increases, the *λ*_max_ blueshifts toward shorter wavelengths. Thus, a higher *E*_T_(30) parameter corresponds to a solvent that is more polar [25,27,37,45]. Figure 7 shows the polarity trends for all the halide systems, which were observed to follow the halide sizes: ChI > ChBr > ChCl > ChF. In each system, the polarity increases with the addition of ChX until it reaches a plateau. Although the final data points for ChI and ChBr are at 10 and 20 mol %, respectively, the data indicate that for all the systems studied, the rate of the polarity increase slows as more salt is introduced. While the ChCl system exhibits higher polarity than ChF, both systems display a similar overall trend.

### 2.5. Self-Diffusion Coefficients

The self-diffusion coefficients of the choline cation, ethylene glycol (EG), and F^−^ were measured for the ChX:EG mixtures at varying salt concentrations using pulsed-field gradient NMR. The results are shown in Table 1. At 10 mol %, the choline diffusion coefficients (×10^−11^ m^2^ s^−1^) increased in the order ChF (4.87) < ChCl (5.77) < ChBr (5.86) < ChI (6.15), while EG showed a similar trend: ChF (7.71) < ChCl (8.59) < ChBr (9.07) < ChI (9.42). In the ChF system, two distinct F^−^ populations were detected at 10 mol %: a slow-diffusing downfield signal (3.90) and a faster upfield signal (7.75). At 20 mol %, the diffusion decreased for both choline and EG in ChCl:EG (4.39 and 7.14, respectively) and ChBr:EG (4.58 and 6.04, respectively). At 33.33 mol % ChF, the diffusion coefficients further decreased to 3.09 (choline), 4.64 (EG), and 2.20 (downfield peak) and 5.29 (upfield peak) for F^−^, indicating reduced self-diffusion for all the species with an increasing salt content.

## 3. Discussion

The transient absorption spectra, with the sample spectra and kinetics shown in Figure 1, reveal the recovery of the ground state (transient bleach peak becomes less negative) as the delay time increases. This can be used to create kinetic decay traces that, when fitted with Equation (1), show biexponential decay behavior yielding two components, *τ*_1_ and *τ*_2_. The solvation dynamics, represented by *τ*_1_ (solvent reorganization response via the EG hydrogen bonding network; Figure 2a) and *τ*_2_ (choline response driven by choline diffusion; Figure 2b), are strongly dependent on the type of halide and the ability to form hydrogen bonds. In the ChF:EG system, the strong hydrogen bonds between F^−^ and the hydroxyl groups of EG create a highly rigid network [21,22,46,47,48]. While F^−^ can form hydrogen bonds with the cation, it preferentially does so with EG [49]. These interactions are reflected in the slower dynamics across all the compositions compared to the ChCl:EG system. For the ChCl:EG systems, the weaker Cl^−^⋯OH hydrogen bonds allow for greater solvent flexibility and faster dynamics compared to ChF. The minima observed at ~16.67 mol % for both *τ*_1_ and *τ*_2_ indicate some similarity between the two systems. Interestingly, in our previous work comparing dry ChF:EG and ChCl:EG at a 1:2 molar ratio, the former exhibited faster dynamics than the latter [21,22]. Here, we report the opposite trend at that composition, which is not very surprising. When there is less than 1 wt % water, the smaller amounts of water present in the mixtures result in slower dynamics [40]. In our previous study, we dried ChF for 72 h and achieved a water content of 500 ppm using Karl Fischer titration [21,22], while here, we dried it for 7 days using the same conditions. It is likely that due to the longer drying times employed in this work, the samples are drier. Comparing the ChCl system to the larger halides, Cl^−^ forms a more compact and structured solvation shell around itself due to its ability to pull electrons more effectively than Br^−^ or I^−^. This results in shorter O–H bond lengths and a denser solvation shell, which should enhance the solvation dynamics and ionic mobility [15]. In addition, the liquidus temperatures (*T*_L_) shown in Figure 3 further illustrate the role of halide-specific interactions. As the halide size increases, the melting point depression becomes less deep. In ChCl:EG, the *T*_L_ reaches a minimum at ~20 mol %, coinciding with the observed dynamic enhancement in the eutectic region, as previously reported in the literature [44]. For ChBr and ChI, the minima are observed at ~10 mol % and ~4 mol %, respectively, but are not accompanied by a dynamic enhancement, as is seen in ChCl. The strength of the hydrogen bonds influences the phase transition temperature, with stronger hydrogen bonding leading to a stronger change in the eutectic properties [50]. This qualitatively explains why in the case of ChI, ChBr, and ChCl, as the anion size decreases and thus the strength of the hydrogen bonding increases [16], there is a deeper suppression of the eutectic liquidus temperature. A similar result was found by van den Bruinhorst et al. in binary mixtures of glutaric acid and tetraethylammonium halides. As the anion size decreased from I^−^ to Cl^−^, the melting point depression became larger. A decreasing anion size strengthens the binary interactions through enhanced hydrogen bonding, demonstrating that the anion is a critical design parameter in tuning DES properties [51]. The ChF system should have the deepest melting point suppression, but there is no observable eutectic point. Instead, the system transitions readily to a supercooled liquid beyond 30 mol % ChF. The similarity to the ChCl system in the *T*_L_ trend and in the dynamical behavior makes it most comparable to the ChCl system, its closest halide neighbor.

In contrast, the dynamics of the ChBr and ChI systems are markedly different than the trends shown in the ChF and ChCl systems. Both the *τ*_1_ and *τ*_2_ generally increase with the salt concentration, with no detectable minimum shown that corresponds to the mildly eutectic composition (~10 mol % for ChBr and ~4 mol % for ChI). The larger size and higher polarizability of Br^−^ and I^−^ weaken their hydrogen-bonding interactions with EG, disrupting the solvent network more significantly than Cl^−^ or F^−^ [15]. This could explain the slower dynamics of the ChBr and ChI systems as the concentration of ChX increases. In addition, the increasing presence of aggregates could contribute to the lack of a dynamics minimum (as is seen at ~16.67 mol % for ChF and ChCl) for ChBr and ChI. This could suggest that rather than forming a well-mixed eutectic phase with enhanced dynamics, these systems may instead exhibit microphase separation or dual-phase behavior, preventing the formation of a eutectic composition with a depressed melting point.

The viscosity, depicted in Figure 4, increases in the order ChCl < ChBr < ChI < ChF. The fact that the ChF system is the most viscous is consistent with the strong hydrogen-bonding interactions between F⁻ and the hydroxyl groups in EG, which lead to higher viscosity [16,21,22,46]. Although ChBr was less viscous than ChI by an average of 1.71 mPa s from 2–10 mol % ChX, the viscosities were very similar [15]. Using larger halides reduces the solvent system’s rigidity, lowering the viscosity compared to ChF. When comparing the ChF and ChCl systems, which have their fastest solvation dynamics at ~16.67 mol % ChX, the increase in viscosity becomes more pronounced after this composition. This correlation suggests that ~16.67 mol % represents a composition for the ChF and ChCl mixtures where the solvent organization and mobility are optimized, as reflected by the observed minimal time constants in the solvation dynamics and the onset of sharper viscosity increases. These observations are consistent with the behavior near the eutectic composition, where the hydrogen-bonding interactions between the salt and solvent components are optimally balanced [44]. Beyond this point, additional salt disrupts this balance, leading to stronger ionic associations, reduced solvent reorganization, and increased viscosity. The ionic conductivity (Figure 5) observed (ChCl > ChBr > ChI > ChF) followed the same trend as the viscosity. For both choline and EG, the experimental self-diffusion coefficients (Table 1) show that, at 10 mol %, ChI > ChBr > ChCl > ChF. Agieienko et al. showed through dielectric relaxation spectroscopy that ChI exhibits a higher ion association constant of ~19 compared to ~10 for ChCl [52], which suggests a stronger tendency for ion pairing or aggregation in the ChI system compared to the ChCl system. Thus, while I^−^ forms weaker hydrogen bonds with EG compared to Cl^−^, I^−^ is more susceptible to ion paring with choline, resulting in fewer free charge carriers being available in the ChI system, despite the high mobility of the remaining free ions. The fast diffusion values in ChI:EG thus reflect the motion of the unbound choline and EG molecules, but the conductivity remains suppressed because of the pairing of some I^−^ species with choline. In the case of the ChBr systems, the choline and EG diffusion are faster than in ChCl, consistent with the weaker hydrogen-bonding interactions of Br^−^. However, the conductivity remains slightly lower in ChBr than in ChCl, which points to the presence of moderate ion pairing or aggregation in ChBr that offsets the increases in mobility.

In addition to the self-diffusion measurements, the trends in the *T*_L_ can also be compared to the viscosity behavior. Notably, the viscosities exhibit sharp increases near the eutectic compositions and beyond. For ChCl, this increase occurs around 15–20 mol %, corresponding to the known eutectic range [44]. In the ChBr system, a similar sharp rise is observed at approximately 10 mol %. Although fewer data points are available for the ChI system, a noticeable viscosity jump occurs near 5 mol % ChI. Interestingly, the ChF system displays two distinct regions of sharp viscosity increase, one between 5 and 10 mol % and another at around 25 mol %. This bimodal behavior may be due to the presence of two distinct solvation environments for F^−^, as evidenced by the two peaks observed in the ^19^F NMR spectrum: one corresponding to F^−^ freely solvated by ethylene glycol and the other to F^−^ associated with choline [22]. See Table 1 and Figure S2.

Factors such as the larger size and polarizability of I^−^ may contribute to a reduced number of free ions or increased ion clustering, limiting the conductivity. The moderately strong Cl^−^⋯OH hydrogen bonds may provide sufficient structuring without making the solvent network overly rigid, allowing for the enhanced mobility of free ions. In contrast, the ChBr and ChI systems display slightly intermediate conductivities. ChF exhibits the lowest conductivity due to the strong F^−^⋯OH hydrogen bonds, which create a more rigid solvent network. From the ^19^F NMR, some of the F^−^ anions are coordinated to choline cations, resulting in fewer free ions. Comparing the diffusion coefficients of all the species in the 10 mol % and 33.33 mol % ChF systems, the diffusion decreases as ChF is added. This trend can be rationalized by the growing number of charge carriers introduced as more ChF is added, which compensates for the reduced mobility of individual ions. Simultaneously, the increase in viscosity and the suppression of diffusion are consistent with the development of a more extensive hydrogen-bonding network, likely reinforced by strong F^−^⋯OH hydrogen bond interactions. In addition, F^−^ has the largest solvodynamic radius, meaning that it is the most hindered of the halides in motion, limiting the free ionic mobility [53]. Cl^−^, Br^−^, and I^−^ have decreasing solvodynamic [53] radii due to the progressively weaker hydrogen bonding, allowing for more freedom of ion mobility. As such, Cl^−^ has the optimal balance between solvodynamic radius and solvated cluster size, leading to its superior ionic conductivity compared to the other halides [15,53].

In general, the density at room temperature (298 K) trend follows ChF < ChCl << ChBr < ChI, with ChF being denser than ChCl at low mol % ChX until 10 mol %. The bigger ChBr and ChI species also show a much more pronounced increase in density as a function of the increasing mol % ChX compared to that of the smaller ChF and ChCl species (Figure 6a). The systems consisting of the bigger halides may be denser due to their larger masses. In contrast, smaller halides form stronger, localized hydrogen bonds with EG with the increasing mol % of ChX (Figure 6b). This may be the cause of the density increase being less pronounced in the ChF and ChCl systems compared to the ChBr and ChI systems. At lower concentrations (2–10 mol %), ChF initially has a higher density than ChCl due to the rigid, tightly packed network formed by the strong F^−^⋯OH hydrogen bonds, which may cause the solvent to “contract” and thus decrease in volume. However, as the concentration increases, the density of ChCl surpasses that of ChF at ~16.67 mol %. At that composition and beyond, the “contracting” nature of the ChF system is likely outweighed by the introduction of the heavier Cl^−^ species in the ChCl system.

The polarity is the result of complex intermolecular interactions between the components of the system, some of which include hydrogen bonding, dipole–dipole forces, dipole–ion interactions, and polarizability [30]. A means to ascertain the solvent polarity is by using a solvatochromic probe like B30, which provides the basis for an empirically derived solvent polarity scale [35]. For all the systems, as ChX salt is added, the *E*_T_(30) polarity increases, as shown in Figure 7. This is likely due to the increase in the ionic species, which enhances the bulk polarity of the systems. However, at higher mol % ChX, the increase in polarity becomes more gradual. The polarity increase with the compositions follows this order: ChI > ChBr > ChCl > ChF. This trend likely correlates with the halide polarizability and the ionic character of their interactions.

## 4. Materials and Methods

### 4.1. Materials

Choline chloride (ChCl) (≥98% purity), ethylene glycol (EG) (anhydrous, ≥99% purity), and Reichardt’s dye betaine-30 (B30) were all purchased from Sigma-Aldrich (St. Louis, MO, USA). Choline bromide (ChBr) (98% purity) and choline iodide (ChI) (98% purity) were purchased from Tokyo Chemical Industry (Chuo-ku, Tokyo, Japan). Silver oxide (98% purity) was purchased from Alfa Aesar (Ward Hill, MA, USA). Hydrofluoric acid (A.C.S. reagent grade, 49% aqueous solution) was purchased from Fisher Scientific (Pittsburgh, PA, USA). See Figure S1 for the chemicals used.

### 4.2. Synthesis of ChF

ChF was synthesized using the procedure reported by Curnow et al. [54]. Choline chloride was converted to choline fluoride by reacting an aqueous choline chloride solution with silver oxide followed by neutralization to pH 7.0 using hydrofluoric acid. The remaining water was removed under a vacuum at 70 °C for 7 days.

### 4.3. Preparation of Solvent Mixtures

Prior to the sample preparation, it was crucial to ensure that all the chemicals used were dry. ChBr and ChI were stored in the glove box (argon atmosphere) upon arrival. ChCl that was not part of the ChF synthesis was dried under a vacuum at 120 °C for 72 h and stored in the glove box. ChF, once neutralized (pH = 7) with hydrofluoric acid, was dried under a vacuum at 70 °C for 7 days and then immediately used for sample preparation. ChCl, ChBr, and ChI were white solids at room temperature (298 K), while ChF remained liquid. EG was dried over a layer of activated molecular sieves for at least 72 h prior to the preparation of the solvent mixtures.

Maintaining a consistent preparation process is essential for ensuring reproducible results. All the ChX solvent systems were prepared by accurately weighing pre-determined amounts of ChF, ChCl, ChBr, and ChI using an analytical balance (±0.0001 g precision). Each salt was then mixed with a measured quantity of EG and subjected to sonication with heating for several hours to support complete dissolution. The resulting stock solutions were clear for ChCl (33.33 mol % ChCl in EG) and ChBr (20 mol % ChBr in EG), slightly yellow for ChI (10 mol % ChI in EG), and brown for ChF (33.33 mol % ChF in EG). Due to the solubility limitations of the ChBr and ChI + EG mixtures at room temperature (298 K), their stock solutions were limited to 20 and 10 mol %, respectively. To obtain mixtures with varying mol % ChX, dilutions were prepared by combining precise amounts of the stock solutions with pure EG. The 0 mol % ChX sample (pure EG) served as a control. For the solvation dynamics and polarity measurements, a small amount of the solvatochromic probe B30 was added to each mixture. Before the measurements were conducted, the samples were carefully sonicated to minimize the heterogeneity.

### 4.4. Differential Scanning Calorimetry

Differential scanning calorimetry (DSC) experiments were performed using a TA Instruments (New Castle, DE, USA) DSC2500 unit from 150 to 350 K. The samples were hermetically sealed in aluminum TZero pans under nitrogen, and data were collected at 2 K min^−1^ ramp rates using heat–cool–heat (HCH) cycles, with replicates. Since these systems exhibit eutectic formation and dual-phase regions, the first-order thermal transition temperatures are herein reported as the liquidus temperatures (*T*_L_ ± 2 K) in lieu of the melting temperatures (*T*_m_). These were determined as the endset of the last discernible melt event coinciding with the highest equilibrium temperature between the solid and liquid phases [55]. As a result, the broad, often-subtle melting behaviors of the dual-phase regions are responsible for the relatively large errors of ±2 K reported in the determination of the *T*_L_. Furthermore, because of the glass transition’s strong dependence on the thermal history and conflating signals from enthalpic relaxation on heating [56,57], the glass transition temperatures (*T*_g_ ± 0.5 K) reported herein are identified as the midpoint of the transition in the reversing heat capacity representation from the temperature-modulated differential scanning calorimetry (TMDSC) measurements.

### 4.5. Femtosecond Transient Absorption Spectroscopy

Femtosecond transient absorption (fs-TA) pump–probe spectroscopy was performed to investigate the solvation dynamics of these mixtures. This technique was used to analyze the solvent response to B30, the solvatochromic probe employed in this study. The use of B30 (optical density of 0.5 per 0.2 cm optical path length) to determine solvent relaxation and kinetics by fs-TA was established by Barbara in the 1990s [39,58,59]. To perform this experiment, a Clark MXR (Dexter, MI, USA) CPA-2010 laser system (780 nm fundamental beam, 130 fs pulse duration, 1 kHz repetition rate, 850 mW output) was used. The laser beam was split into two paths: one was frequency-doubled to 390 nm using a *β*-barium borate crystal for the pump beam, while the other generated a broadband white-light supercontinuum (450–800 nm) with a sapphire crystal for the probe beam. A computer-controlled optical delay stage introduced time delays for measuring the differential absorption spectra. Both the steady-state and fs-TA measurements were conducted in a 2 mm glass cuvette at room temperature and ambient pressure. The steady-state absorption spectra, recorded before and after the fs-TA experiments, confirmed that no photodegradation of B30 occurred. Optical chirp correction was applied using Surface Xplorer (version 4.3.0) software, while biexponential fitting and data analysis were performed in Microcal Origin (version 9.7.5.184) to ensure all the errors were kept within ±10%.

### 4.6. Steady-State Absorption Spectroscopy and Solvent Polarity

Steady-state absorption measurements were conducted on a Varian (Santa Clara, CA, USA) Cary 50 UV-Vis spectrophotometer to analyze the shifts in the visible CT absorption band maximum of B30 (see Figure S1) in response to variations in the solvent polarity, with a maximum error of ±1 nm. B30 exhibits negative solvatochromism, with the CT absorption band shifting to shorter wavelengths (blueshift) in more polar environments. The solvent polarity was evaluated using Reichardt’s *E*_T_(30) scale, which leverages the pronounced solvatochromism of B30 [27,28,30,31,33,45]. For the steady-state absorption measurements, each B30-enriched sample was baselined against its corresponding sample without B30 to ensure accurate spectral analysis.

### 4.7. Viscosity and Density

The viscosity (*η*) of the mixtures was measured using a Rheosense (San Ramon, CA, USA) microVISC viscometer, with approximately 100 µL of each sample introduced in a temperature-controlled environment at 298 K. The viscosity measurements were performed in triplicate, averaged, and reported with the associated uncertainties. The density (*ρ*) was determined using an Anton Paar (Graz, Styria, Austria) DMA-5000 density meter, calibrated with degassed deionized water, and measured at 5 K increments from 298.15 to 323.15 K, with a standard deviation of ≤±0.5 K. The density meter was calibrated using degassed deionized water at 293 K and the maximum standard deviation for the calibration reference was 5 × 10^−5^ g mL^−1^.

### 4.8. Ionic Conductivity

The ionic conductivity (*σ*) of the samples at 298 K was measured using a Thermo Scientific (Waltham, MA, USA) Orion Star A222 conductivity meter equipped with a 2-electrode conductivity cell (measurement range: 10 to 2000 mS cm^−1^). The instrument was calibrated using three standard sodium chloride solutions with conductivities of 0.100, 1.413, and 12.9 mS cm^−1^. Each sample was measured in triplicate, and the results were averaged, with the uncertainties included in the reported data and in the SI.

### 4.9. NMR Spectroscopy and Pulsed-Field Gradient (PFG) NMR

All the NMR experiments were carried out on a Bruker (Billerica, MA, USA) Avance 400 spectrometer operating at a magnetic field strength of 9.4 T, equipped with a direct observe BBFO 5 mm z-gradient probe. The samples were prepared under an argon atmosphere in a glovebox and flame-sealed in 5 mm NMR tubes to prevent exposure to air and moisture. The experiments were performed at 298.2 K after allowing 15 min for thermal equilibration. The pulsed-field gradient (PFG) NMR measurements were performed to determine the self-diffusion coefficients of selected species. The experiments were conducted in the ^1^H domain (for Ch⁺ and EG) and the ^19^F domain (for F^−^) using the bipolar pulse longitudinal eddy current delay (BPP-LED) pulse sequence. Each experiment consisted of 8 transients per increment, using 16,384 points in the F2 dimension, with spectral widths of 14 ppm for ^1^H and 16 ppm for ^19^F. The relaxation delay was set to at least five times the T_1_, and four dummy scans were applied prior to the acquisition. The gradient strengths were incremented linearly from 2% to 95% of the maximum value (50 G cm^−1^) over 16 steps. For each experiment, the diffusion time (Δ) was fixed at 0.2 s and the gradient pulse duration (*δ*) was optimized within the 4–6 ms range to achieve approximately 95% signal attenuation for the slowest-diffusing species at the final gradient step. The raw spectra were subjected to manual phasing and automatic baseline correction. The data were processed using an exponential filter in the F2 dimension with a line broadening (LB) of 0.3 Hz. The signal integrals were fitted to the Stejskal–Tanner [60] equation, Equation (3), using the T1/T2 module in Bruker TopSpin:

(3) $$\frac{I}{I_{0}}=e^{\left[-D\gamma^{2}g^{2}\delta^{2}\left(∆-\frac{\delta }{3}\right)\right]}\;$$

In Equation (3), *I* is the signal intensity with the gradient applied, *I*_0_ is the initial signal intensity, *γ* is the gyromagnetic ratio of the investigated nucleus, and *g* is the gradient strength applied. The uncertainty in the determined self-diffusion coefficients was below 5%.

The one-dimensional (1D) NMR spectra of ^19^F, ^79^Br, and ^127^I were acquired to investigate the chemical environments of the halide species. The ^19^F NMR spectrum was acquired on the neat sample, while the ^79^Br and ^127^I spectra were recorded from 20% *v*/*v* solutions in D_2_O. The ^19^F NMR (resonance frequency: 376.5 MHz) was acquired with a spectral width of 16 ppm, 8 scans, a relaxation delay of 8 s, and an FID size of 8192 points. The data were processed with a line broadening (LB) of 3 Hz. The ^79^Br NMR (resonance frequency: 134.3 MHz) was acquired with a spectral width of 246 ppm, using 8192 scans, a relaxation delay of 5 s, and an FID size of 1024 points. The data were processed with an LB of 30 Hz. The ^127^I NMR (resonance frequency: 107.7 MHz) was acquired with a spectral width of 400 ppm, using 32,768 scans, a relaxation delay of 10 s, and an FID size of 1024 points. The data were processed with an LB of 100 Hz. All the spectra were processed using Bruker TopSpin (version 4.3.0) software, including manual phasing and automatic baseline correction. See Figures S2–S4 for the NMR spectra.

## 5. Conclusions

This study demonstrates that the halide size and hydrogen bond strength significantly impact the solvation dynamics, viscosity, conductivity, density, and polarity of the ChX:EG eutectic solvent systems. Smaller halides (F^−^, Cl^−^) form stronger hydrogen bonds with EG, creating more rigid networks and slower dynamics, while larger halides (Br^−^, I^−^) weaken the hydrogen bonding, disrupting the solvent structure, and still lead to progressively slower dynamics due to their heavier mass. As the halide size increases, the melting point depression becomes less deep. While ChF is expected to show the greatest melting point suppression, no clear eutectic point is observed. Instead, the system transitions to a supercooled liquid beyond 30 mol % ChF. Its *T*_L_ and dynamical trends most closely resemble those of ChCl, its nearest halide analog. The viscosity follows the trend of ChCl < ChBr < ChI < ChF, while the conductivity follows the inverse order, with Cl^−^ balancing hydrogen bonding and ion mobility most effectively. The viscosity trends also align with the *T*_L_ behavior, showing sharp increases near the eutectic compositions. Notably, ChF exhibits two distinct viscosity jumps, likely due to the presence of two solvation environments for F^−^, free-solvated in EG and choline-associated, which is supported by the ^19^F NMR. The density trends show ChF is initially denser than ChCl at a low mol %, but the trend reverses beyond ~16.67 mol %, with Br^−^ and I^−^ contributing to the more pronounced density increases. The *E*_T_(30) polarity measurements confirm that adding ChX salts increases the bulk solvent polarity, though the increase slows at a higher mol %. The polarity trend (ChI > ChBr > ChCl > ChF) aligns with the halide size and polarizability. These findings inform the selection of anions to tune the properties of eutectic solvent mixtures and may aid in the rational design of future electrolyte systems with tailored properties.

## Figures and Tables

**Table 1:** Experimentally determined self-diffusion coefficients measured using PFG NMR at 298 K for choline (Ch^+^) and ethylene glycol (EG) in the ChF:EG (10 mol %), ChCl:EG (10 mol %), ChBr:EG (10 mol %), ChI:EG (10 mol %), ChCl:EG (20 mol %), ChBr:EG (20 mol %), and ChF:EG (33.33 mol %) systems. The ChF:EG systems also show fluoride (^19^F) diffusion. The ChF:EG (33.33 mol %) system is from Alfurayj et al. [22] The uncertainty in the determined self-diffusion coefficients was below 5%. See Figures S2–S4 for the corresponding NMR spectra.

System	Self-Diffusion Coefficient (×10^−11^ m^2^ s^−1^)		
	Ch^+^	EG	^19^F
ChF:EG (10 mol %)	4.87	7.71	3.90 (downfield peak); 7.75 (upfield peak)
ChCl:EG (10 mol %)	5.77	8.59	-
ChBr:EG (10 mol %)	5.86	9.07	-
ChI:EG (10 mol %)	6.15	9.42	-
ChCl:EG (20 mol %)	4.39	7.14	-
ChBr:EG (20 mol %)	4.58	6.04	-
ChF:EG (33.33 mol %) [22]	3.09	4.64	2.20 (downfield peak); 5.29 (upfield peak)

## Data Availability

The original contributions presented in this study are included in the article/Supplementary Material. Further inquiries can be directed to the corresponding author.

## Supplementary Materials

The following supporting information can be downloaded at https://www.mdpi.com/article/10.3390/molecules30102113/s1, Figure S1: Representative molecular structures illustrate the laser-pulse-induced intramolecular charge transfer (CT) process in B30, transitioning from its zwitterionic ground state to its radicalized excited state upon photoexcitation. The structures of the various choline halides and ethylene glycol (EG) are also shown; Figure S2: ^19^F NMR spectra, FWHM—full width at half maximum: 0.02 ppm (7Hz) and 0.08 ppm (28.5 Hz). Neat sample; Figure S3: ^79^Br NMR spectra, FWHM—full width at half maximum: 32 ppm (3.2 kHz). The sample was diluted to 20% *v*/*v* in D_2_; Figure S4: ^127^I NMR spectra, FWHM—full width at half maximum: 90 ppm (9 kHz). The sample was diluted to 20% *v*/*v* in D_2_O; Table S1: Maximum CT band absorption wavelength ($\lambda_{abs}^{max})$, *E*_T_(30) polarity, and dynamics (*τ*_1_, fast; *τ*_2_, slow; *τ*_avg_, average) measurements for ChF:EG at various mol % ChF. *T* = 298 K; Table S2: Maximum CT band absorption wavelength ($\lambda_{abs}^{max})$, *E*_T_(30) polarity, and dynamics (*τ*_1_, fast; *τ*_2_, slow; *τ*_avg_, average) measurements for ChCl:EG at various mol % ChCl. *T* = 298 K; Table S3: Maximum CT band absorption wavelength ($\lambda_{abs}^{max})$, *E*_T_(30) polarity, and dynamics (*τ*_1_, fast; *τ*_2_, slow; *τ*_avg_, average) measurements for ChBr:EG at various mol % ChBr. *T* = 298 K; Table S4: Maximum CT band absorption wavelength ($\lambda_{abs}^{max})$, *E*_T_(30) polarity, and dynamics (*τ*_1_, fast; *τ*_2_, slow; *τ*_avg_, average) measurements for ChI:EG at various mol % ChI. *T* = 298 K; Table S5: Numerical values of the viscosity (*η*) and ionic conductivity (*σ*) of the ChF:EG mixtures at varying mol % ChF. *T* = 298 K; Table S6: Numerical values of the viscosity (*η*) and ionic conductivity (*σ*) of the ChCl:EG mixtures at varying mol % ChCl. *T* = 298 K; Table S7: Numerical values of the viscosity (*η*) and ionic conductivity (*σ*) of the ChBr:EG mixtures at varying mol % ChBr. *T* = 298 K; Table S8: Numerical values of the viscosity (*η*) and ionic conductivity (*σ*) of the ChI:EG mixtures at varying mol % ChI. *T* = 298 K; Table S9: Densities (*ρ*) of the ChF:EG mixtures at varying mol % ChF and temperatures; Table S10: Densities (*ρ*) of the ChCl:EG mixtures at varying mol % ChCl and temperatures; Table S11: Densities (*ρ*) of the ChBr:EG mixtures at varying mol % ChBr and temperatures; Table S12: Densities (*ρ*) of the ChI:EG mixtures at varying mol % ChI and temperatures.
