# Supplementary material for: Tuning Solvation Dynamics of Electrolytes at Their Eutectic Point Through Halide Identity
Source: Molecules. 2025 May 9;30(10):2113. doi: 10.3390/molecules30102113 (PMC12113864; doi:10.3390/molecules30102113)
Supplement: Supplementary file 1 [file molecules-30-02113-s001.zip › molecules-3607081-supplementary.pdf]

# Supplementary Information

# Tuning Solvation Dynamics of Electrolytes at their Eutectic Point through Halide Identity

**Rathiesh Pandian 1, Benworth B. Hansen 2, Giselle de Araujo Lima e Souza 3, Joshua R. Sangoro 2, Steven Greenbaum 3 and Clemens Burda 1,***

1 Department of Chemistry, College of Arts and Sciences, Case Western Reserve University, Cleveland, OH 44106, USA

2 William G. Lowrie Department of Chemical and Biomolecular Engineering, The Ohio State University, Columbus, OH 43210, USA

3 Department of Physics & Astronomy, Hunter College of the City University of New York, New York, NY 10065, USA

* Correspondence: burda@case.edu

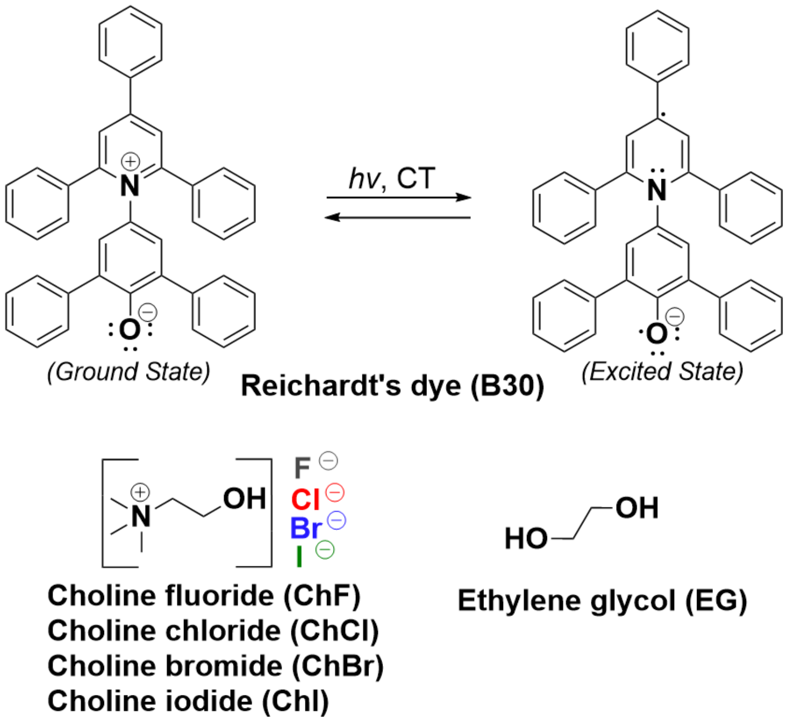


**Figure S1.** Representative molecular structures illustrate the laser pulse-induced intramolecular charge transfer (CT) process in B30, transitioning from its zwitterionic ground state to its radicalized excited state upon photoexcitation. The structures of the various choline halides and ethylene glycol (EG) are also shown.

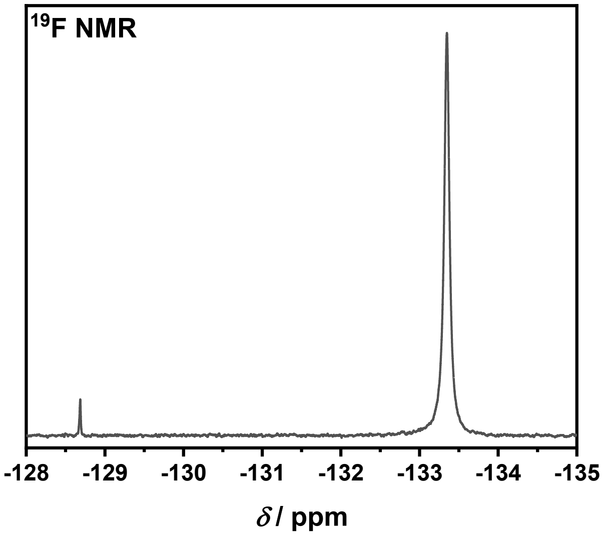


**Figure S2:** $^{19}$F NMR spectra of 10 mol % ChF in EG, FWHM – Full Width at Half Maximum: 0.02 ppm (7Hz) and 0.08 ppm (28.5 Hz). Neat sample.

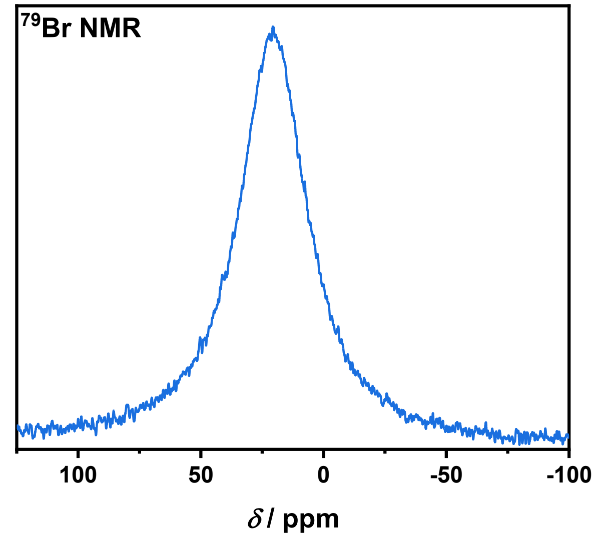


**Figure S3:** $^{79}$Br NMR spectra of 10 mol % ChBr in EG, FWHM – Full Width at Half Maximum: 32 ppm (3.2 kHz). The sample was diluted to 20% v/v in $D_2O$.

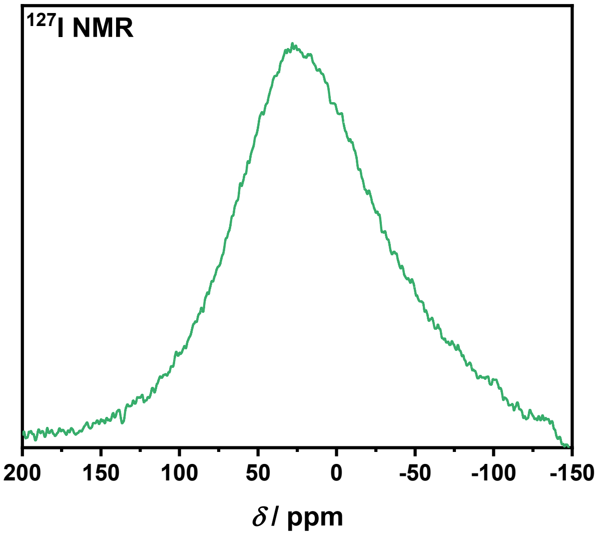


**Figure S4:** $^{127}$I NMR spectra of 20 mol % ChI in EG, FWHM – Full Width at Half Maximum: 90 ppm (9 kHz). The sample was diluted to 20% v/v in $D_2O$.

**Table S1.** Maximum CT band absorption wavelength ($\lambda_{max}^{abs}$) of Reichardt's dye B30, $E_T(30)$ polarity, and dynamics ($\tau_1$, fast; $\tau_2$, slow; $\tau_{avg}$, average) measurements for ChF:EG at various mol % ChF. $T$ = 298 K.

| mol % ChF | $\lambda_{max}^{abs}$ (nm) | $E_T(30)$ (kcal mol$^{-1}$) | $\tau_1$(ps) | $\tau_2$ (ps) | $\tau_{avg}$ (ps) |
|---|---|---|---|---|---|
| 0 | 511.00 | 55.95 | 13.4 ± 0.1 | 138 ± 3.3 | 31.3 |
| 2 | 506.66 | 56.43 | 20.0 ± 0.4 | 325 ± 20.4 | 41.6 |
| 5 | 504.97 | 56.62 | 18.7 ± 0.5 | 295 ± 10.7 | 34.7 |
| 10 | 504.52 | 56.67 | 17.5 ± 0.6 | 241 ± 10.2 | 46.2 |
| 16.67 | 503.19 | 56.82 | 16.2 ± 0.8 | 156 ± 9.7 | 39.7 |
| 20 | 503.10 | 56.83 | 17.8 ± 0.6 | 196 ± 17.8 | 45.1 |
| 25 | 502.74 | 56.87 | 19.3 ± 0.4 | 233 ± 20.7 | 55.0 |
| 33.33 | 502.21 | 56.93 | 21.9 ± 0.8 | 291 ± 6.50 | 67.4 |

**Table S2.** Maximum CT band absorption wavelength ($\lambda_{max}^{abs}$) of Reichardt's dye B30, $E_T(30)$ polarity, and dynamics ($\tau_1$, fast; $\tau_2$, slow; $\tau_{avg}$, average) measurements for ChCl:EG at various mol % ChCl. $T$ = 298 K.

| mol % ChCl | $\lambda_{max}^{abs}$ (nm) | $E_T(30)$ (kcal mol$^{-1}$) | $\tau_1$(ps) | $\tau_2$ (ps) | $\tau_{avg}$ (ps) |
|---|---|---|---|---|---|
| 0 | 511.00 | 55.95 | 13.4 ± 0.1 | 138 ± 3.3 | 31.3 |
| 2 | 503.96 | 56.73 | 19.0 ± 0.5 | 246 ± 11.0 | 49.6 |
| 5 | 500.84 | 57.09 | 16.9 ± 0.1 | 200 ± 10.0 | 43.1 |
| 10 | 499.58 | 57.23 | 13.9 ± 0.1 | 135 ± 8.6 | 30.7 |
| 16.67 | 498.62 | 57.34 | 12.8 ± 0.2 | 107 ± 5.8 | 29.3 |
| 20 | 498.36 | 57.37 | 14.3 ± 0.1 | 127 ± 7.1 | 25.5 |
| 25 | 497.93 | 57.42 | 17.0 ± 0.1 | 179 ± 6.5 | 31.2 |
| 33.33 | 497.49 | 57.47 | 20.5 ± 0.1 | 239 ± 10.3 | 43.0 |

**Table S3.** Maximum CT band absorption wavelength ($\lambda_{max}^{abs}$) of Reichardt's dye B30, $E_T(30)$ polarity, and dynamics ($\tau_1$, fast; $\tau_2$, slow; $\tau_{avg}$, average) measurements for ChBr:EG at various mol % ChBr. $T$ = 298 K.

| mol % ChBr | $\lambda_{max}^{abs}$ (nm) | $E_T(30)$ (kcal mol$^{-1}$) | $\tau_1$(ps) | $\tau_2$ (ps) | $\tau_{avg}$ (ps) |
|---|---|---|---|---|---|
| 0 | 511.00 | 55.95 | 13.4 ± 0.1 | 138 ± 3.3 | 31.3 |
| 2 | 504.06 | 56.72 | 13.7 ± 0.4 | 111 ± 7.6 | 22.8 |
| 5 | 499.97 | 57.19 | 16.4 ± 0.3 | 138 ± 11.5 | 29.1 |
| 10 | 494.06 | 57.87 | 19.7 ± 0.3 | 201 ± 15.6 | 42.5 |
| 16.67 | 491.94 | 58.12 | 22.2 ± 0.3 | 252 ± 11.7 | 57.8 |
| 20 | 491.03 | 58.23 | 22.7 ± 0.5 | 260 ± 19.4 | 60.7 |

**Table S4.** Maximum CT band absorption wavelength ($\lambda_{max}^{abs}$) of Reichardt's dye B30, $E_T(30)$ polarity, and dynamics ($\tau_1$, fast; $\tau_2$, slow; $\tau_{avg}$, average) measurements for ChI:EG at various mol % ChI. $T$ = 298 K.

| mol % ChI | $\lambda_{max}^{abs}$ (nm) | $E_T(30)$ (kcal mol$^{-1}$) | $\tau_1$(ps) | $\tau_2$ (ps) | $\tau_{avg}$ (ps) |
|---|---|---|---|---|---|
| 0 | 511.00 | 55.95 | 13.4 ± 0.1 | 138 ± 3.3 | 31.3 |
| 2 | 499.97 | 57.19 | 18.2 ± 0.3 | 100 ± 4.8 | 33.7 |
| 5 | 493.00 | 57.99 | 19.5 ± 0.7 | 147 ± 10.4 | 43.8 |
| 10 | 489.96 | 58.35 | 20.0 ± 0.5 | 164 ± 10.6 | 42.1 |

**Table S5.** Numerical values of viscosity ($\eta$) and ionic conductivity ($\sigma$) of ChF:EG mixtures at varying mol % ChF. $T$ = 298 K.

| mol % ChF | $\eta$ (mPa s) | $\sigma$ (mS cm$^{-1}$) |
|---|---|---|
| 0 | 19.07 ± 0.1 | 0.012 ± 0.002 |
| 2 | 26.00 ± 0.1 | 0.790 ± 0.035 |
| 5 | 26.61 ± 0.2 | 1.742 ± 0.016 |
| 10 | 29.76 ± 0.8 | 3.224 ± 0.038 |
| 16.67 | 35.78 ± 0.3 | 4.941 ± 0.004 |
| 20 | 38.49 ± 1.0 | 5.637 ± 0.060 |
| 25 | 41.93 ± 0.6 | 6.457 ± 0.017 |
| 33.33 | 64.08 ± 0.8 | 7.348 ± 0.033 |

**Table S6.** Numerical values of viscosity ($\eta$) and ionic conductivity ($\sigma$) of ChCl:EG mixtures at varying mol % ChCl. $T$ = 298 K.

| mol % ChCl | $\eta$ (mPa s) | $\sigma$ (mS cm$^{-1}$) |
|---|---|---|
| 0 | 19.07 ± 0.1 | 0.012 ± 0.002 |
| 2 | 19.27 ± 1.0 | 1.768 ± 0.004 |
| 5 | 19.65 ± 0.1 | 3.081 ± 0.010 |
| 10 | 20.33 ± 0.1 | 5.988 ± 0.024 |
| 16.67 | 24.23 ± 0.3 | 9.386 ± 0.066 |
| 20 | 26.21 ± 0.4 | 9.551 ± 0.050 |
| 25 | 31.92 ± 0.1 | 9.481 ± 0.009 |
| 33.33 | 53.00 ± 1.0 | 8.745 ± 0.006 |

**Table S7.** Numerical values of viscosity ($\eta$) and ionic conductivity ($\sigma$) of ChBr:EG mixtures at varying mol % ChBr. $T$ = 298 K.

| mol % ChBr | $\eta$ (mPa s) | $\sigma$ (mS cm$^{-1}$) |
|---|---|---|
| 0 | 19.07 ± 0.1 | 0.012 ± 0.002 |
| 2 | 21.22 ± 0.1 | 1.534 ± 0.077 |
| 5 | 23.93 ± 0.2 | 3.228 ± 0.093 |
| 10 | 26.33 ± 0.6 | 5.963 ± 0.006 |
| 16.67 | 30.48 ± 0.3 | 7.964 ± 0.062 |
| 20 | 34.34 ± 0.6 | 8.481 ± 0.076 |

**Table S8.** Numerical values of viscosity ($\eta$) and ionic conductivity ($\sigma$) of ChI:EG mixtures at varying mol % ChI. $T$ = 298 K.

| mol % ChI | $\eta$ (mPa s) | $\sigma$ (mS cm$^{-1}$) |
|---|---|---|
| 0 | 19.07 ± 0.1 | 0.012 ± 0.002 |
| 2 | 23.73 ± 0.1 | 1.448 ± 0.005 |
| 5 | 24.82 ± 0.3 | 3.203 ± 0.090 |
| 10 | 28.06 ± 0.8 | 5.451 ± 0.035 |

**Table S9.** Densities ($\rho$) of ChF:EG mixtures at varying mol % ChF and temperatures. Calibration of the density meter was achieved using degassed deionized water at 293 K and the maximum standard deviation for the calibration reference was 5 × $10^{-5}$ g mL$^{-1}$.

| mol % ChF | $\rho$ (g mL$^{-1}$) | | | | | |
|---|---|---|---|---|---|---|
| | 298.15 K | 303.15 K | 308.15 K | 313.15 K | 318.15 K | 323.15 K |
| 0 | 1.110140 | 1.106540 | 1.103020 | 1.099480 | 1.095920 | 1.092320 |
| 2 | 1.112276 | 1.108847 | 1.105381 | 1.101903 | 1.098399 | 1.094870 |
| 5 | 1.112362 | 1.108662 | 1.105245 | 1.101805 | 1.098351 | 1.094879 |
| 10 | 1.112711 | 1.109449 | 1.106172 | 1.102863 | 1.099537 | 1.096196 |
| 16.67 | 1.113240 | 1.110056 | 1.106853 | 1.103603 | 1.100236 | 1.096702 |
| 20 | 1.113616 | 1.110504 | 1.107373 | 1.104227 | 1.101061 | 1.097871 |
| 25 | 1.113964 | 1.110373 | 1.107345 | 1.104362 | 1.101383 | 1.098376 |
| 33.33 | 1.114509 | 1.111636 | 1.108740 | 1.105833 | 1.102902 | 1.099882 |

**Table S10.** Densities ($\rho$) of ChCl:EG mixtures at varying mol % ChCl and temperatures. Calibration of the density meter was achieved using degassed deionized water at 293 K and the maximum standard deviation for the calibration reference was 5 × $10^{-5}$ g $mL^{-1}$.

| mol % ChCl | $\rho$ (g $mL^{-1}$) | | | | | |
|---|---|---|---|---|---|---|
| | 298.15 K | 303.15 K | 308.15 K | 313.15 K | 318.15 K | 323.15 K |
| 0 | 1.110140 | 1.106540 | 1.103020 | 1.099480 | 1.095920 | 1.092320 |
| 2 | 1.110500 | 1.107070 | 1.103640 | 1.100190 | 1.096730 | 1.093250 |
| 5 | 1.111400 | 1.108010 | 1.104600 | 1.101180 | 1.097740 | 1.094290 |
| 10 | 1.112350 | 1.109060 | 1.105760 | 1.102460 | 1.099140 | 1.095810 |
| 16.67 | 1.113830 | 1.110740 | 1.107670 | 1.104620 | 1.101550 | 1.098470 |
| 20 | 1.114280 | 1.111200 | 1.108120 | 1.105040 | 1.101960 | 1.098880 |
| 25 | 1.114930 | 1.111940 | 1.108950 | 1.105970 | 1.102990 | 1.100000 |
| 33.33 | 1.116530 | 1.113720 | 1.110910 | 1.108100 | 1.105310 | 1.102530 |

**Table S11.** Densities ($\rho$) of ChBr:EG mixtures at varying mol % ChBr and temperatures. Calibration of the density meter was achieved using degassed deionized water at 293 K and the maximum standard deviation for the calibration reference was 5 × $10^{-5}$ g $mL^{-1}$.

| mol % ChBr | $\rho$ (g $mL^{-1}$) | | | | | |
|---|---|---|---|---|---|---|
| | 298.15 K | 303.15 K | 308.15 K | 313.15 K | 318.15 K | 323.15 K |
| 0 | 1.110140 | 1.106540 | 1.103020 | 1.099480 | 1.095920 | 1.092320 |
| 2 | 1.123922 | 1.120459 | 1.116963 | 1.113449 | 1.109921 | 1.106378 |
| 5 | 1.141600 | 1.138133 | 1.134655 | 1.131165 | 1.127656 | 1.124129 |
| 10 | 1.168615 | 1.165216 | 1.161783 | 1.158335 | 1.154878 | 1.151413 |
| 16.67 | 1.206661 | 1.203270 | 1.199882 | 1.196494 | 1.193101 | 1.189721 |
| 20 | 1.224611 | 1.221180 | 1.218015 | 1.214643 | 1.211261 | 1.207986 |

**Table S12.** Densities ($\rho$) of ChI:EG mixtures at varying mol % ChI and temperatures. Calibration of the density meter was achieved using degassed deionized water at 293 K and the maximum standard deviation for the calibration reference was 5 × $10^{-5}$ g $mL^{-1}$.

| mol % ChI | $\rho$ (g $mL^{-1}$) | | | | | |
|---|---|---|---|---|---|---|
| | 298.15 K | 303.15 K | 308.15 K | 313.15 K | 318.15 K | 323.15 K |
| 0 | 1.110140 | 1.106540 | 1.103020 | 1.099480 | 1.095920 | 1.092320 |
| 2 | 1.137133 | 1.133592 | 1.130013 | 1.126447 | 1.122874 | 1.119315 |
| 5 | 1.172585 | 1.169020 | 1.165440 | 1.161851 | 1.158245 | 1.154632 |
| 10 | 1.231946 | 1.228450 | 1.224864 | 1.221261 | 1.217639 | 1.214006 |
